# Predictors of Microvascular Reperfusion After Myocardial Infarction

**DOI:** 10.1007/s11886-021-01442-1

**Published:** 2021-02-23

**Authors:** Daniel J. Doherty, Robert Sykes, Kenneth Mangion, Colin Berry

**Affiliations:** 1 West of Scotland Regional Heart and Lung Centre, Golden Jubilee National Hospital, Glasgow, UK; 2 British Heart Foundation Glasgow Cardiovascular Research Centre, Institute of Cardiovascular and Medical Sciences, University of Glasgow, Glasgow, UK; 3 Department of Cardiology, Queen Elizabeth University Hospital, NHS Greater Glasgow and Clyde Health Board, Glasgow, UK

**Keywords:** Microvascular obstruction, Myocardial infarction, MRI, Stratified medicine

## Abstract

**Purpose of Review:**

In acute ST-segment elevation myocardial infarction (STEMI), successful restoration of blood flow in the infarct-related coronary artery may not secure effective myocardial reperfusion. The mortality and morbidity associated with acute MI remain significant. Microvascular obstruction (MVO) represents failed microvascular reperfusion. MVO is under-recognized, independently associated with adverse cardiac prognosis and represents an unmet therapeutic need.

**Recent Findings:**

Multiple factors including clinical presentation, patient characteristics, biochemical markers, and imaging parameters are associated with MVO after MI.

**Summary:**

Impaired microvascular reperfusion is common following percutaneous coronary intervention (PCI). New knowledge about disease mechanisms underpins precision medicine with individualized risk assessment, investigation, and stratified therapy. To date, there are no evidence-based therapies to prevent or treat MVO post-MI. Identifying novel therapy for MVO is the next frontier.

## Introduction

Acute ST-segment elevation myocardial infarction (STEMI) is a major cause of premature death and morbidity [1]. Restoration of epicardial coronary blood flow by PCI is the evidence-based standard of care [2, 3]. Significant progress has been made in terms of reducing time to revascularization over the last two decades [4]. Despite this, figures for short-term mortality remain unchanged and the incidence of chronic heart failure post-MI is high [2, 5].

Microvascular reperfusion is the ability to perfuse the coronary microcirculation in a previously ischemic region after opening the epicardial vessel. Successful restoration of epicardial blood flow does not guarantee myocardial reperfusion. There is evidence of impaired microvascular perfusion on cardiac magnetic resonance (CMR) imaging in over half of patients following STEMI despite angiographic patency of the infarct-related artery post-PCI [6]. This acute and potentially reversible complication is known as microvascular obstruction (MVO) and is thought to occur post-coronary reperfusion as a result of endothelial disruption and microvascular thrombi [7]. In the absence of adequate reperfusion, progression to irreversible myocardial hemorrhage can occur at the infarct core [8]. MVO and subsequent intramyocardial hemorrhage (IMH) are strongly associated with mortality and hospitalization for heart failure [9•].

In clinical practice, MVO is likely to pass undetected in many cases. The limits of contemporary coronary revascularization in known myocardial ischemia have been highlighted in the recent ISCHEMIA trial [10]. The focus of reperfusion therapy in STEMI is extending to consider the preservation of distal coronary microvasculature, in turn contemporary practice guidelines call for research to identify new treatments for MVO [2]. An understanding of the predictors of microvascular reperfusion after MI is central to the development of investigative pathways and trial design and lays the foundations for much needed targeted therapeutic intervention and stratified medicine in this cohort.

In this review, we consider the definition of microvascular reperfusion after MI on a practical basis in a range of clinical settings throughout the patient journey. We provide an overview of the predictors of coronary microvascular dysfunction in this context and address the concept of stratified medicine in the future.

## Defining Microvascular Reperfusion After Myocardial Infarction—a Practical Approach

### Pathophysiology and Natural History

Coronary microvasculature holds 20% of myocardial blood volume and modulates myocardial perfusion [11, 12]. Impaired microvascular reperfusion is a complex pathophysiological process that stems from initial embolic phenomena and the resultant ischemic sequelae of edema, hemorrhage, and intracellular substance release. This results in luminal obstruction, external compression, and endothelial dysfunction with impaired vasomotion [13–15]. In the event of inadequate reperfusion, chronic hibernation and cell death occur leading to the development of ischemic cardiomyopathy and clinical heart failure [16].

The evolution of microvascular reperfusion after MI is relevant when considering assessment methods used to define it. Experimental models indicate that myocardial perfusion defects occur during the process of reperfusion [17]. Radiological evidence of MVO in humans is maximal at an early stage (4–12 h post-reperfusion), remains stable to day two and then decreases [6]. By 1-month post-reperfusion, MVO has reversed in 50% of patients, and in the majority of cases, there is complete resolution after 8 months [18, 19]. IMH is a manifestation of severe microvascular injury that only occurs in the context of MVO and exhibits a progressive time course to a peak at 2 days [20].

Coronary microvascular dysfunction runs a dynamic temporal course following reperfusion in acute MI. The definition of microvascular reperfusion is dependent on method of detection and also the time point at which assessment takes place. Impaired microvascular reperfusion defined using different methods is prognostically important [9•, 21•, 22].

MVO occurs following reperfusion in both STEMI and non-ST-elevation acute myocardial infarction (NSTEMI). NSTEMI is a heterogeneous condition with a variable clinical course that typically exhibits a relapsing and remitting myocardial ischemia that is less clamant than is the case for STEMI. Rates of MVO following early revascularization in NSTEMI are lower [23, 24] but nonetheless associated with adverse prognosis [25]. Similar mechanisms underpin MVO in both conditions and there may be shared predictive factors. The majority of research evaluating the prevalence and prognostic impact of MVO has been undertaken in STEMI populations, and this provides a more reliable basis for the definition and prediction of microvascular reperfusion after MI.

### Clinical Setting

**Fig. 1:**
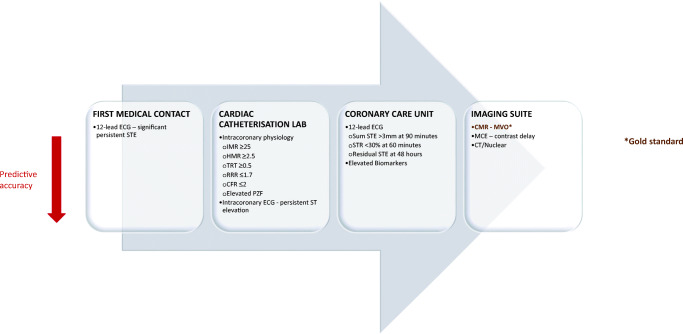
Markers of impaired microvascular reperfusion after STEMI—investigation hierarchy during the patient journey

The test options for assessing reperfusion injury differ according to the time-point and location in the patient care pathway (Fig. 1).

### Cardiac Catheterization Laboratory—Coronary Angiography

#### Visual Angiographic Assessment

The thrombolysis in myocardial infarction (TIMI) flow grading system is a widely used visual assessment of flow appearance that provides an immediate evaluation of microvascular reperfusion [26]. Complete antegrade flow following intervention is denoted as “TIMI 3” and is associated with improved survival compared to reduced flow grades [27]. There is evidence of impaired microcirculation in the majority of patients with significantly reduced TIMI grade [9•]; therefore, the angiographic definition of MVO is TIMI grade < 2. TIMI grade does not correlate well with MVO defined by “gold standard” CMR imaging [28, 29]. Multiple studies have reported MVO in more than half of those with optimal TIMI grade 3 flow following PCI [6, 28, 30].

Methods such as myocardial blush grading (MBG) and fluoroscopy assisted scoring have been introduced to enhance visual assessment and reduce operator variability [31, 32]. MBG grade 3 indicates successful microvascular reperfusion, whereas grades 0–1 define MVO in this setting. While these techniques are intended to improve classification, correlation with MVO is only moderate, and these techniques are not relied upon in clinical practice [33–35].

The spatial resolution of coronary angiography is approximately tenfold larger than the diameter of arterioles that govern myocardial blood flow [36]. Visual angiographic assessment remains an inaccurate means to define microvascular reperfusion following STEMI and has a limited role.

#### Intracoronary Electrocardiogram (IC-ECG)

An intracoronary unipolar electrocardiogram can be acquired while in the catheterization laboratory by connecting the proximal end of the angioplasty guidewire to a monitor using a crocodile clip [37]. IC-ECG is more sensitive than conventional 12-lead ECG at detecting regional myocardial ischemia and has the added benefit of real-time information with no precordial leads that can obscure angiographic images [38]. Resolution of ST-elevation (STR) defines successful microvascular reperfusion in this context and correlates with improved survival after STEMI [39, 40]. STR demonstrates predictive value for MVO at 4 days post-STEMI but lacks specificity [41].

#### Invasive Coronary Physiology

Coronary physiology measurements using diagnostic guidewire sensors have emerged as useful tools in the diagnosis and management of disorders of coronary function [36]. These metrics provide an immediate assessment of microvascular resistance and can be used to define microvascular reperfusion post-PCI. There are a number of parameters that are relevant in this context (Table 1).

**Table 1:** Defining microvascular reperfusion by invasive coronary physiology after MI

Physiology metric	Measurement	Indicates impaired microvascular reperfusion	Predictive accuracy vs. IMR	Comment
IMR	Minimum microvascular resistance at peak hyperemia	Elevated (≥ 25)	–	Highest predictive accuracy for MVO
HMR	Distal coronary pressure divided by mean Doppler flow velocity	Elevated (≥ 2.5)	Similar	Complex technique
TRT	Time for temperature at the guidewire tip to return to baseline during maximal hyperemia	Elevated (≥ 0.5)	Similar	Limited use in clinical practice
CFR	Epicardial and microcirculatory vasodilator capacity derived from Doppler or thermodilution	Reduced (≤ 2)	Reduced	Broad measure with low predictive accuracy
RRR	Difference between basal resting tone in the microcirculation and resistance at maximal hyperemia	Reduced (≤ 1.7)	Reduced	–
PZF	Index derived from an extrapolation of coronary pressure-flow velocity loops	Elevated	Unclear	–

##### Index of Microcirculatory Resistance (IMR)

IMR represents the minimum microvascular resistance during adenosine-induced hyperemia and is a validated measure of microcirculatory reperfusion [21•, 42]. Low IMR values indicate successful microvascular reperfusion, whereas raised values (≥ 25) suggest MVO and are associated with increased rates of heart failure hospitalization and major adverse cardiovascular events (MACE) [21•, 30, 43, 44]. Current evidence suggests that IMR is the metric with the highest predictive accuracy for MVO and IMH. In the BHF MR-MI study, an IMR of > 27 had the highest diagnostic accuracy for MVO (0.65) and myocardial hemorrhage (0.68) revealed by CMR 2 days post-MI [30]. Furthermore, a higher IMR (e.g., > 40) corresponds less with CMR findings and more with all-cause death and heart failure [21•, 45]. These findings imply a gradient of prognostic significance: an IMR ≥ 25 is the upper limit of the reference range, an IMR of 28 corresponds with microvascular pathology that may be reversible, i.e., microvascular obstruction, and an IMR of 40 or higher is most closely associated with adverse cardiovascular events. This is our interpretation of the clinical significance of the different IMR thresholds which hopefully clarifies the utility of this tool when considering its role in clinical practice [46].

##### Hyperemic Microvascular Resistance (HMR)

HMR is calculated by dividing distal coronary pressure by mean Doppler flow velocity at peak hyperemia. In keeping with IMR, low values indicate successful microvascular reperfusion whereas raised values (≥ 2.5) are suggestive of MVO [47]. Predictive accuracy is comparable to IMR although there is less empirical evidence and real-world application may be limited by technique complexity.

##### Coronary Flow Reserve (CFR)

CFR reflects epicardial *and* microcirculatory vasodilator capacity and can be derived utilizing Doppler or thermodilution techniques [48]. High CFR readings indicate successful microcirculatory reperfusion and low values (≤ 2) correlate with MVO, although this is not a consistent trend in the literature [30]. CFR is a broad measure and lacks the predictive accuracy and reproducibility to quantify microvascular reperfusion after MI.

##### Resistive Reserve Ratio (RRR)

RRR is a measure of the difference between basal resting tone in the microcirculation and resistance at maximal hyperemia and reflects the ability of the coronary microcirculation to vary resistance to hyperemic stimuli. Elevated values indicate increased vasodilator capacity and thus successful microvascular reperfusion. Reduced RRR (≤ 1.7) is associated with the presence and extent of MVO, albeit to a lesser extent than IMR when using dichotomized values [21•, 49, 50].

##### Temperature Recovery Time (TRT)

TRT is the time taken for the temperature at the guidewire tip to return to baseline during maximal hyperemia. Low TRT indicates successful microcirculatory reperfusion. Recent research demonstrates correlation between MVO and longer recovery time (TRT ≥ 0.5) with similar predictive accuracy to IMR [51].

##### Coronary Pressure-Flow Velocity Loops

During mid-diastole, a linear relationship exists between pressure and flow. Coronary zero flow pressure (PZF) is an index derived from the extrapolation of coronary pressure-flow velocity loops [52]. Theoretically this represents the intraluminal pressure required to maintain vessel patency in the absence of coronary flow, i.e., against extra-vascular compressive forces only. Low PZF indicates successful microcirculatory reperfusion, and raised values are predictive of CMR-defined MVO following STEMI (AUC 0.75, 95% CI 0.55–0.89; *p* = 0.01) [47]. PZF is predictive of infarct size and myocardial viability [53, 54].

## Cardiovascular Imaging Options

### Transthoracic Echocardiography (TTE)

Echocardiography is a standard care assessment in patients following an acute MI. Reduced ejection fraction carries prognostic significance and is more common in populations with MVO [6, 55]. Regional wall motion abnormality is a surrogate for infarct size, which correlates positively with MVO [9•]. Although readily available and useful for risk stratification, standard echocardiography lacks the specificity to define successful microvascular reperfusion after MI.

The coronary flow-velocity ratio (CFVR) is derived from diastolic flow measurements in epicardial coronary arteries during rest/stress using Doppler echocardiography. In the absence of epicardial flow limitation, CFVR > 2.5 indicates normal microvascular function [11, 56]. To our knowledge, this technique has not been validated against CMR-defined MVO in major studies. Speckle-tracking techniques for strain assessment in 2D and 3D models demonstrate significant association with CMR-defined MVO in small studies [57, 58]. Myocardial contrast echo (MCE) uses ultrasound to visualize micro-bubble contrast in the myocardium following intravenous injection. Contrast signal intensity is a surrogate for myocardial blood flow and successful microvascular reperfusion is indicated by rapid and uniform myocardial contrast enhancement. Contrast delay or the absence of myocardial contrast opacification suggests MVO [59, 60]. This approach has demonstrated utility in the identification of MVO as a negative prognostic marker [22, 61]. When compared to CMR in a canine model, both techniques correlate well with MVO histologically, although CMR was found to have increased sensitivity in reduced blood flow states [62]. Clinical use of MCE is limited by operator dependence, cost, advanced skill requirement, and limited spatial resolution.

### Cardiac Magnetic Resonance Imaging (CMR)

Regional myocardial ischemia results in edema (increased water content) and prolongs longitudinal (T1) and transverse (T2) relaxation times increasing regional signal intensity on CMR images [63]. MVO is defined as a lack of gadolinium enhancement (hypointense core) within hyper-enhanced infarct areas [62].

Contrast-enhanced CMR is considered the gold-standard in vivo assessment of MVO and is the reference diagnostic test in trials that have uncovered the prognostic implications of impaired microvascular reperfusion following MI [9•, 64]. CMR has been validated histologically in this context and its value is increasingly recognized in terms of characterizing infarct pathology as well as providing accurate structural and functional myocardial assessment [8, 65].

### Other Imaging Modalities

Delayed contrast-enhanced CT has potential for tissue characterization; however, unlike CMR, CT is limited by image quality and radiation exposure [66].

Nuclear imaging is frequently used to assess myocardial blood flow, and positron emission tomography (PET) is a validated means of coronary vasomotor function testing. Myocardial scintigraphy can detect capillary reperfusion defects following coronary thrombolysis [67]. The role of these techniques in defining microvascular reperfusion following MI is less clear and accessibility out-with main centers is limited.

## Clinical Risk Factors for Failed Microvascular Reperfusion After Myocardial Infarction

**Fig. 2:**
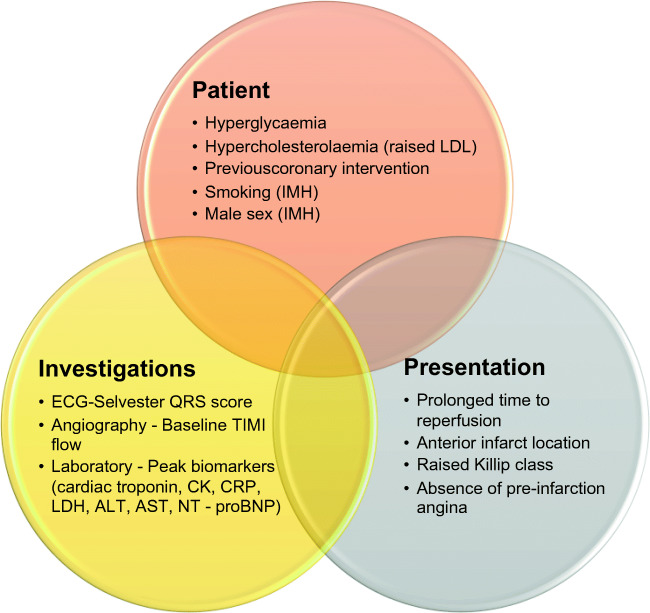
Predictive factors associated with MVO after STEMI

Well-recognize factors linked to adverse cardiac prognosis may confer their detrimental effects via microvascular obstruction. Underlying health conditions, clinical presentation features, ECG parameters, and laboratory results have a role in predicting the likelihood of successful microvascular reperfusion after MI (Fig. 2). Microvascular obstruction on CMR is the gold-standard assessment of impaired microvascular reperfusion and represents the benchmark comparison when reviewing predictive factors unless otherwise stated.

## Cardiovascular Risk Factors

Cardiovascular risk factors are common in patients presenting with STEMI. Comorbid conditions, such as hypertension and diabetes, are associated with endothelial dysfunction [68] and could signify underlying microvascular disease that influences the extent of microvascular reperfusion following MI.

### Diabetes, Hyperglycemia, and Obesity

Diabetes has established multisystemic macro- and microvascular complications. The condition increases the probability of a cardiovascular event and worsens prognosis thereafter [69]. Hyperglycemia exerts a detrimental effect on endothelial cell function and impairs arterial vasodilation [70]. In the setting of acute MI, poor glycemic control is associated with worse cardiac outcomes [71].

Large studies have been unable to demonstrate convincing association between pre-existing diabetes and MVO following re-perfused STEMI. There does however appears to be a link between hyperglycemia at presentation and subsequent microvascular dysfunction following PCI [72, 73]. In 93 patients who underwent CMR at 7 days following reperfusion, admission glucose level was an independent predictor of MVO (OR 1.014, 95% CI 1.004–1.023; *p* = 0.006) [74]. This relationship exists even in non-diabetic populations [75]. To date, MVO has proven resistant to therapeutic modification [76, 77].

Obesity (defined by raised body mass index) has not been shown to demonstrate significant correlation with microvascular perfusion defects post-STEMI [30, 78].

### Hypertension

Hypertension is associated with endothelial dysfunction and is a strong negative prognostic indicator after STEMI [79–81]. However, studies utilizing both CMR and invasive coronary physiology have been unable to demonstrate significant association between pre-existing hypertension and microvascular dysfunction following acute MI [79, 82].

### Hypercholesterolemia

Hypercholesterolemia is associated with coronary heart disease and cholesterol-lowering therapy conveys mortality benefit in this cohort [83]. A degree of controversy exists surrounding the value of admission cholesterol levels and clinical outcomes following STEMI [84, 85]. Animal models using histological diagnosis demonstrate increased MVO in the context of coronary reperfusion after a cholesterol-enriched diet [86]. Associations between hypercholesterolemia and MVO following re-perfused MI in humans are less clear. Reindl et al. reported admission low-density lipoprotein (LDL) levels to be an independent predictor of MVO in over 200 revascularized STEMI patients (OR 1.02, 95% CI 1.01–1.02; *p* = 0.002) [87]. Other clinical trials using CMR- and MCE-based diagnostic assessments did not report significant differences in patients with normal versus raised cholesterol levels [30, 88].

### Smoking

Cigarette smoking negatively impacts coronary endothelial function and increases the risk of developing cardiovascular disease [89, 90]. There is limited evidence to suggest that smoking is associated with MVO post-STEMI and links between smoking and IMH are yet to be clearly defined [30, 91–93]. Haig et al. report evidence of *improved* microvascular perfusion defined by coronary physiology and ECG parameters in the acute phase after reperfusion in smokers, followed by subsequent development of microvascular injury. Ultimately, when adjusted for infarct size, cigarette smoking was identified as an independent predictor for IMH (OR 2.76, 95% CI 1.42–3.77; *p* = 0.003) but not MVO [94].

## Non-modifiable Patient Characteristics

### Age

Elderly STEMI patients who have undergone revascularization are more likely to demonstrate impaired microvascular reperfusion defined by ECG STR and angiographic TIMI grade [93, 95–97]. Large contemporary studies examining microvascular reperfusion following STEMI using the more robust measure of CMR-defined MVO have not demonstrated clear association with age [9•, 30]. A small trial examining predictive factors in 97 patients following revascularized STEMI found that MVO was more prevalent in *younger* patients [29]. These findings are not supported elsewhere in the literature.

### Sex

Females presenting with STEMI, especially when under the age of 60, have a higher mortality rate than males [98–100]. This has been attributed to atypical symptoms (leading to delayed presentation and treatment) as well as a possible increased prevalence of microvascular disease [101, 102]. The impact of cardiac risk factors on mortality is sex-dependent; e.g., diabetes is more likely to be associated with endothelial dysfunction in females [100]. Significant associations between female sex and markers of impaired microvascular reperfusion after STEMI have not been consistently reported [103]. In fact, prospective studies have shown correlation between male sex and IMH in the acute period following reperfusion [6, 30].

STEMI predominately affects men who present at a younger age with a first MI than is the case for women. This fact highlights natural sex differences in the epidemiology of ischemic heart disease.

### Genetic Susceptibility

Genetic variants contribute to an individual’s risk of coronary artery disease and single-nucleotide polymorphisms have been identified within certain genes (VEGFA, CDKN2B-AS1) that correlate with microvascular dysfunction [104, 105]. Research in this area is preliminary and based on microvascular function defined by coronary flow reserve in patient populations not restricted to myocardial infarction. The applicability of these findings in predicting microvascular reperfusion after MI is limited.

## Clinical Presentation

### Electrocardiography (ECG)

The 12-lead ECG is the standard-of-care diagnostic assessment in myocardial infarction. The extent of STR has been used as measure of epicardial reperfusion dating back to the era of cardiac thrombolysis. Variations of this approach now form part of contemporary assessment of microvascular dysfunction after revascularized STEMI where complete STR indicates successful microcirculatory reperfusion and failed STR (< 30% resolution) or persistent ST-elevation (STE) despite adequate flow appearance is attributed to MVO. ST-elevation is greater in individuals with MVO before and after coronary intervention [106]. There are conflicting results regarding the utility of STR in detecting MVO [107, 108]. As an alternative measure, residual STE in either a single lead (maximum ST-segment deviation) or combined leads has been associated with CMR-defined MVO to a greater extent than STR. Husser et al. demonstrated that sum STE > 3 mm 90 min post-PCI is an independent predictor of MVO (OR 3.1, 95% CI 1.2–8.4; *p* = 0.02). Other studies report correlation between residual STE and MVO at 48–72 h [106, 108, 109].

QRS duration on presentation has been shown to be independently associated with angiographic no-reflow (OR 1.07, 95% CI 1.02–1.12; *p* = 0.003) [110]. The Selvester QRS score incorporates multiple criteria in different leads relating to QRS amplitude and duration [111]. It is a validated tool for predicting the extent of myocardial damage in STEMI, and recent studies using CMR demonstrate independent association (OR 1.362, 95% CI 1.038–1.951; *p* = 0.024) and predictive value for MVO (AUC 0.64, 95% CI 0.56–0.72; *p* = 0.001) [112, 113]. There is no clear association reported between the presence and extent of pathological Q waves and MVO development following PCI [108, 114].

### Time to Reperfusion

Reducing “door-to-balloon” time improves mortality in STEMI [115]. Treatment delay in broader terms from symptom onset to reperfusion seems to increase the likelihood of MVO. For over 40 years, there has been recognized association between microvascular dysfunction and myocardial ischemic time. This was first demonstrated by Kloner in 1974 where the microvascular effects of varying coronary occlusion times were compared in dogs and defined using electron microscopy. Only prolonged occlusion (90 min vs. 40 min) resulted in extensive microvascular damage and edema [116]. A large meta-analysis examined this more closely in revascularized STEMI patients. MVO was encountered more frequently in those with delayed reperfusion in this cohort, in particularly “symptom to device” time (symptom onset until beginning of reperfusion procedure) (*p* = < 0.0001) [9•]. This is in agreement with previous research in this area [29, 93, 96].

### Coronary Lesion Specifics and Clinical Features

The development of MVO is associated with larger infarct size, anterior infarct location, and reduced initial TIMI grade [6, 9•]. Individuals with previous coronary intervention are at increased risk of developing IMH following reperfusion [6]. Previous saphenous vein coronary bypass grafting was reported to increase risk of angiographic no-reflow defined as TIMI grade 0–1 [117]. Using the same measure in a revascularized STEMI cohort, target coronary lesion length and thrombus burden are independent predictors of microvascular dysfunction [93, 96, 97].

Killip class (extent of heart failure at presentation with MI) is significantly linked with MVO across a range of assessment methods [6, 29, 93, 97]. The absence of angina pre-infarction has also been identified as an independent predictor of MVO following PCI (OR 8.35, 95% CI 1.27–54.71; *p* = 0.027) [114, 118], and this observation has stimulated interest in whether pre-, post- [119], or remote conditioning might be a novel therapy to prevent MVO. To date, clinical trial results have not supported this possibility [120].

### Biomarkers

Plasma levels of cardiac troponin are associated with the presence and extent of MVO following re-perfused STEMI. Various cut-off values have been proposed that demonstrate high predictive accuracy, particularly if using peak levels [121–125]. Peak troponin levels have superior predictive value compared to ECG scoring parameters (AUC 0.81, 95% CI 0.75–0.87; *p* = <0.001 vs. AUC 0.64, 95% CI 0.56–0.72; *p* = 0.001) [112] and considering other biomarkers in addition to troponin does not seem to increase MVO predictive value [121].

Elevated circulating N-terminal pro-brain natriuretic peptide (NTpro-BNP) also demonstrates association with MVO in this population [126]. In a 2013 study, levels remained significantly elevated in those with MVO complicated by IMH at each time point over a 3-month period following coronary revascularization [125].

Other biomarkers that have demonstrated association with the development of MVO post re-perfused STEMI include creatinine kinase (CK), high-sensitivity C-reactive protein (hsCRP), lactate dehydrogenase (LDH), aspartate aminotransferase (AST), and alanine aminotransferase (ALT)—suggesting a possible link to inflammatory conditions. Mayr et al. demonstrated significantly elevated CK, hsCRP, and LDH within the first 4 days following PCI in those later confirmed to have MVO. These markers all correlated with MVO size [123]. A CMR follow-up study supported these findings in addition to linking MVO with significantly elevated levels of AST and ALT in revascularized MI. In this study, all biochemical parameters shared similar statistical significance in terms of predicting microvascular dysfunction (AUC 0.68–0.79) aside from NT-proBNP which was weakly lower (AUC 0.64, *p* = <0.05). Although peak values where more prognostically useful, sensitivity and specificity for MVO did not exceed 80% [121].

### Predictive Scores

An evolving understanding of factors that are associated with MVO after MI has led to the development of predictive scoring systems. Husser et al. compared clinical parameters, ECG, biomarker, and angiographic data in the prediction of impaired microvascular reperfusion following STEMI. Their score includes Killip class (double weighting), age < 55, diabetes, time to reperfusion, and sum STE. Incidence of MVO increased with each score increment and is reported as 93% for scores ≥ 3 (*n* = 44, *p* = < 0.001). Predictive value was superior to residual STE or angiographic parameters and similar to peak troponin levels [29].

Pre-existing scoring systems have been applied to the assessment of microvascular dysfunction after acute MI. The SYNTAX score uses a combination of coronary characteristics from angiography. Magro et al. demonstrated the utility of this scoring system as an independent predictor of angiographic no-reflow in 669 patients after revascularized STEMI (OR 1.29, 95% CI 1.02–1.63; *p* = <0.001) [127, 128]. The CHA_2_DS_2_-VASc score is a widely used predictor of thromboembolic events in atrial fibrillation. When applied retrospectively to a STEMI population, it has also been shown to have predictive value for angiographic no-reflow (OR 1.58, 95% CI 1.33–1.88; *p* = < 0.001) [129, 130].

## Clinical Relevance and Therapeutic Options for Impaired Microvascular Reperfusion After MI

Coronary microvascular disease is not benign. Impaired microvascular reperfusion joins established markers of adverse cardiac prognosis such as infarct size [131]. In a meta-analysis investigating microvascular injury in 1025 patients following STEMI, MVO was an independent predictor of major adverse cardiac events HR 3.74, 95% CI 2.21–6.64; *p* = < 0.001) [64]. This is in concordance with results from a pooled analysis that included 1688 patients undergoing CMR within 7 days following STEMI. Hazard ratio for all-cause mortality was 1.09 (95% CI 1.01–1.17; *p* = 0.03) and demonstrated a graded increase dependent on the extent of MVO as a percentage of left ventricular myocardial mass [9•].

The significance of MVO in patients with acute MI has spurred research in recent years into the investigation of adjunctive therapies that aim to modify MVO and improve outcomes. Potential interventions include intracoronary drug delivery (fibrinolytics and vasodilator therapy), deferred coronary stenting, ischemic conditioning, parenteral beta-blockade, and antiplatelets among other pharmacotherapies. To date, major trial data relating to these methods has largely failed to demonstrate consistent improvements to either markers of microvascular reperfusion or MACE.

The METOCARD-CNIC randomized control trial investigated the effect of intravenous metoprolol administered prior to reperfusion in anterior STEMI with Killip class ≤ 2. Metoprolol use was associated with a 40% reduction in the extent of MVO on CMR at 1 week. This remained significant even when adjusted for infarct size and other confounding factors. Infarct size and left-ventricular ejection fraction were improved at 6 months in the metoprolol group but incidence of MACE at 2 years was comparable [132, 133]. Previous trials investigating beta-blocker therapy prior to coronary revascularization did not demonstrate benefit in terms of MACE or infarct size at 30 days [134].

Studies are underway that are investigating the impact of novel interventions including pressure-controlled intermittent coronary sinus occlusion (NCT03625869), localized intracoronary hypothermia (NCT03447834), selective strategies to defer stenting (NCT01542385) [135], and outcomes associated with stratified therapy based on non-invasive imaging in the management of ischemic heart disease [136].

## Conclusion

The mortality and morbidity associated with acute MI remain significant. Impaired microvascular reperfusion is a common complication following coronary intervention in this condition. This is defined as MVO and can be detected at different time-points across a range of modalities. MVO is under-recognized, independently associated with adverse cardiac prognosis, and represents an unmet therapeutic need.

A range of factors have emerged that correlate with MVO after MI. An enhanced understanding of these predictive factors forms the foundation for a move towards precision medicine with individualized risk assessment, investigation, and subsequent stratified therapy with novel treatments. The successful modification of this process represents the next therapeutic frontier.
